# The design and evaluation of gamified online role-play as a telehealth training strategy in dental education: an explanatory sequential mixed-methods study

**DOI:** 10.1038/s41598-024-58425-9

**Published:** 2024-04-22

**Authors:** Chayanid Teerawongpairoj, Chanita Tantipoj, Kawin Sipiyaruk

**Affiliations:** 1 Department of Advanced General Dentistry, Faculty of Dentistry, Mahidol University, Bangkok, Thailand; 2 Department of Orthodontics, Faculty of Dentistry, Mahidol University, Bangkok, Thailand

**Keywords:** Dental education, Distance learning, Game-based learning, Gamification, Role-play, Telehealth, Health care, Dentistry, Health services, Public health

## Abstract

To evaluate user perceptions and educational impact of gamified online role-play in teledentistry as well as to construct a conceptual framework highlighting how to design this interactive learning strategy, this research employed an explanatory sequential mixed-methods design. Participants were requested to complete self-perceived assessments toward confidence and awareness in teledentistry before and after participating in a gamified online role-play. They were also asked to complete a satisfaction questionnaire and participate in an in-depth interview to investigate their learning experience. The data were analyzed using descriptive statistics, paired sample t-test, one-way analysis of variance, and framework analysis. There were 18 participants who completed self-perceived assessments and satisfaction questionnaire, in which 12 of them participated in a semi-structured interview. There were statistically significant increases in self-perceived confidence and awareness after participating in the gamified online role-play (*P* < 0.001). In addition, the participants were likely to be satisfied with this learning strategy, where usefulness was perceived as the most positive aspect with a score of 4.44 out of 5, followed by ease of use (4.40) and enjoyment (4.03). The conceptual framework constructed from the qualitative findings has revealed five key elements in designing a gamified online role-play, including learner profile, learning settings, pedagogical components, interactive functions, and educational impact. The gamified online role-play has demonstrated its potential in improving self-perceived confidence and awareness in teledentistry. The conceptual framework developed in this research could be considered to design and implement a gamified online role-play in dental education. This research provides valuable evidence on the educational impact of gamified online role-play in teledentistry and how it could be designed and implemented in dental education. This information would be supportive for dental instructors or educators who are considering to implement teledentistry training in their practice.

## Introduction

Telehealth has gained significant attention from various organization due to its potential to improve healthcare quality and accessibility^1^. It can be supportive in several aspects in healthcare, including medical and nursing services, to enhance continuous monitoring and follow-up^2^. Its adoption has increased substantially during the COVID-19 pandemic, aiming to provide convenient healthcare services^3^. Even though the COVID-19 outbreak has passed, many patients still perceive telehealth as an effective tool in reducing a number of visits and enhancing access to health care services^4,5^. This supports the use of telehealth in the post-COVID-19 era.

Teledentistry, a form of telehealth specific to dentistry, has been employed to improve access to dental services^6^. This system offers benefits ranging from online history taking, oral diagnosis, treatment monitoring, and interdisciplinary communication among dental professionals, enabling comprehensive and holistic treatment planning for patients^7^. Teledentistry can also reduce travel time and costs associated with dental appointments^8–10^. There is evidence that teledentistry serves as a valuable tool to enhance access to dental care for patients^11^. Additionally, in the context of long-term management in patients, telehealth has contributed to patient-centered care, by enhancing their surrounding environments^12^. Therefore, teledentistry should be emphasized as one of digital dentistry to enhance treatment quality.

Albeit the benefits of teledentistry, available evidence demonstrates challenges and concerns in the implementation of telehealth. Lack of awareness and knowledge in the use of telehealth can hinder the adoption of telehealth^13^. Legal issues and privacy concerns also emerge as significant challenges in telehealth use^14^. Moreover, online communication skills and technology literacy, including competency in using technological tools and applications, have been frequently reported as challenges in teledentistry^15,16^. Concerns regarding limitations stemming from the lack of physical examination are also significant^17^. These challenges and complexities may impact the accuracy of diagnosis and the security and confidentiality of patient information. Therefore, telehealth training for dental professionals emerges as essential prerequisites to effectively navigate the use of teledentistry, fostering confidence and competence in remote oral healthcare delivery.

The feasibility and practicality of telehealth in dental education present ongoing challenges and concerns. Given the limitations of teledentistry compared to face-to-face appointments, areas of training should encompass the telehealth system, online communication, technical issues, confidentiality concerns, and legal compliance^18^. However, there is currently no educational strategy that effectively demonstrates the importance and application of teledentistry^19^. A role-play can be considered as a teaching strategy where learners play a role that closely resembles real-life scenarios. A well-organized storytelling allows learner to manage problematic situations, leading to the development of problem-solving skill^20,21^. When compared to traditional lecture-based learning, learners can also enhance their communication skills through conversations with simulated patients^22,23^. In addition, they could express their thoughts and emotions during a role-play through experiential learning^20,24,25^. Role-play through video teleconference would be considered as a distance learning tool for training dental professionals to effectively use teledentistry.

While there have been studies supporting online role-play as an effective learning tool due to its impact of flexibility, engagement, and anonymity^26,27^, no evidence has been yet reported whether or not this learning strategy could have potential for training teledentistry. Given the complicated issues in telehealth, role-play for training teledentistry should incorporate different learning aspects compared to face-to-face communication with patients. In addition, game components have proved to be supportive in dental education^28,29^. Consequently, this research aimed to evaluate user perceptions and educational impact of gamified online role-play to enhance learner competence and awareness in using teledentistry as well as to construct a conceptual framework highlighting how to design and implement this interactive learning strategy. This research would introduce and promote the design and implementation of gamified online role-play as a learning tool for training teledentistry. To achieve the aim, specific objectives were established as follows:

- 1. To design a gamified online role-play for teledentistry training.
- 2. To investigate learner perceptions regarding their confidence and awareness in the use of teledentistry after completing the gamified online role-play.
- 3. To explore user satisfactions toward the use of gamified online role-play.
- 4. To develop a conceptual framework for designing and implementing a gamified online role-play for teledentistry training.

## Materials and methods

### Research design

This research employed an explanatory sequential mixed-methods design, where a quantitative phase was firstly performed followed by a qualitative phase^30,31^. The quantitative phase was conducted based on pre-experimental research using one-group pretest–posttest design. Participants were requested to complete self-perceived assessments toward confidence and awareness in the use of teledentistry before and after participating in a gamified online role-play. They were also asked to complete a satisfaction questionnaire in using a gamified online role-play for training teledentistry. The qualitative phase was afterwards conducted to explore in-depth information through semi-structured interviews, in order to enhance an understanding of the quantitative phase, and to develop a conceptual framework for designing and implementing an online role-play for training teledentistry.

### A gamified online role-play for training teledentistry

A gamified online role-play was designed and developed by the author team. To ensure its educational impact was significant, the expected learning outcomes were formulated based on insights gathered from a survey with experienced instructors from the Department of Advanced General Dentistry, Faculty of Dentistry, Mahidol University. These learning outcomes covered areas of online communication skill, technical issues, technology literacy of patients, limitations of physical examination, and privacy concerns of personal information. Learning scenario and instructional content were subsequently designed to support learners in achieving the expected learning outcomes, with their alignments validated by three experts in dental education. A professional actress underwent training to role-play a patient with a dental problem, requesting a virtual consultation or teledentistry. Before conducting data collection, the simulated patient was required to undergo a training and adjusting process with a pilot group under supervision of two experts in advanced general dentistry and dental education who had experience with teledentistry to ensure realism and completeness of learning content.

According to the role-play scenario, an actress was assigned to portray a 34-year-old female with chief complaints of pain around both ears, accompanied by difficulties in chewing food due to tooth loss. She was instructed to express her anxiety and nervousness about addressing these issues. Additionally, it was specified that she could not take a day off from work during this period. Despite this constraint, she required a dental consultation to receive advice for initial self-care, as her symptoms significantly impacted her daily life. Furthermore, she was designated to encounter difficulties with the technological use of the teledentistry platform.

The game components were implemented into the online role-play to enhance motivation and engagement. As challenge and randomness appear to be game elements^32,33^, five challenge cards were designed and embedded into the online role-play, where a participant was asked to randomly select one of them before interacting with the simulated patient. The challenging situations were potential technical concerns which could occur frequently during video conferencing, including network problems (e.g., internet disconnection and poor connection) and audiovisual quality issues. The participants were blinded to the selected card, while it was revealed to only the simulated patient. The challenging conditions were mimicked by the organizers and simulated patient, allowing learners to deal with difficulties. Therefore, both challenges and randomness were implemented into this learning intervention not only to create learning situations but also to enhance engagement.

A feedback system was carefully considered and implemented into the gamified online role-play. Immediate feedback appears to be a key feature of interactive learning environments^29^. Formative feedback was instantly delivered to learners through verbal and non-verbal communication, including words (content), tone of voice, facial expressions, and gestures of the simulated patient. This type of feedback allowed participants to reflect on whether or not their inputs were appropriate, enabling them to learn from their mistakes, or so-called the role of failure^34^. Summative feedback was also provided at the end of the role-play through a reflection from a simulated patient and suggestions from an instructor.

**Figure 1:**
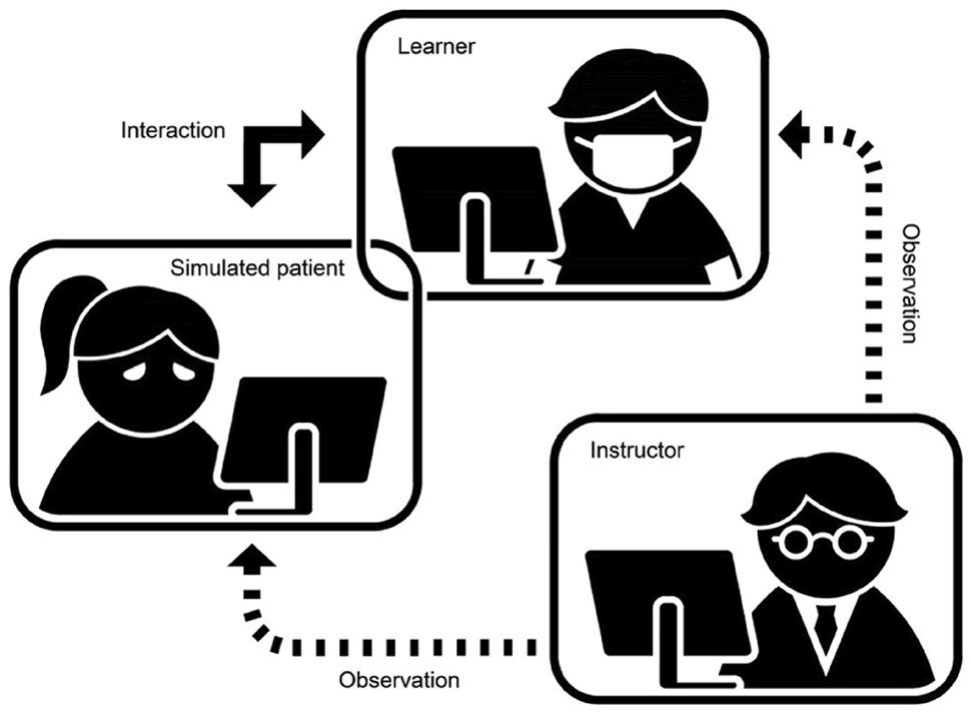
A diagram demonstrating the setting of gamified online role-play.

Learners were able to interact with the simulated patient using an online meeting room by Cisco WebEx. According to the research setting (Fig. 1), a learner was asked to participate in the role-play activity using a computer laptop in a soundproof room, while a simulated patient was arranged in a prepared location showing her residential environment. The researcher and instructor also joined the online meeting room and observed the interaction between the simulated patient and learners during the role-play activity whether or not all necessary information was accurately obtained. The role-play activity took around 30 minutes.

### Research participants

#### Quantitative phase

The participants in this research were postgraduate students from the Residency Training Program in Advanced General Dentistry at Mahidol University Faculty of Dentistry in academic year 2022, using a volunteer sampling. This program was selected because its objective was to develop graduates capable of integrating competencies from various dental disciplines to provide comprehensive dental care for both normal patients and those with special needs. Therefore, teledentistry should be a supportive component of their service. The recruitment procedure involved posting a recruiting text in the group chat of the residents. Those interested in participating in the research were informed to directly contact us to request more information, and they were subsequently allowed to decide whether they would like to participate. This approach ensured that participation was voluntary. Although there could be a non-response bias within this non-probability sampling technique^35^, it was considered as appropriate for this study, as participants were willing to have contribution in the learning activity, and therefore accurate and reliable research findings with no dropout could be achieved^36^.

The inclusion and exclusion criteria were established to determine the eligibility of prospective participants for this research. This study included postgraduate students from Years 1 to 3 in the Residency Training Program in Advanced General Dentistry at Mahidol University Faculty of Dentistry, enrolled during the academic year 2022. They were also required to at least complete the first semester to be eligible for this research to ensure familiarity with comprehensive dental care. However, they were excluded if they had previous involvement in the pilot testing of the gamified online role-play or if they were not fluent in the Thai language. The sample size was determined using a formula for two dependent samples (comparing means)^37^. To detect a difference in self-perceived confidence and awareness between pre- and post-assessments at a power of 90% and a level of statistical significance of 1%, five participants were required. With an assumed dropout rate of 20%, the number of residents per year (Year 1–3) was set to be 6. Therefore, 18 residents were required for this research.

#### Qualitative phase

The participants from the quantitative phase were selected for semi-structured interviews using a purposive sampling. This sampling method involved the selection of information-rich participants based on specific criteria deemed relevant to the research objective and to ensure a diverse representation of perspectives and experiences within the sample group^38^. In this research, the information considered for the purposive sampling included demographic data (e.g., sex and year of study), along with self-perceived assessment scores. By incorporating perceptions from a variety of participants, a broad spectrum of insights from different experiences in comprehensive dental practice and diverse improvement levels in self-perceived confidence and awareness could inform the design and implementation of the training program effectively. The sample size for this phase was determined based on data saturation, wherein interviews continued until no new information or emerging themes were retrieved. This method ensured thorough exploration of the research topic and maximized the richness of the qualitative data obtained.

### Outcome assessments

To evaluate the gamified online role-play, a triangular design approach was employed, enabling the researchers to compare the research outcomes from different assessment methods. In this research, self-perceived assessments (confidence and awareness) in teledentistry, satisfactions toward gamified online role-play, and learner experience were assessed to assure the quality and feasibility of the gamified online role-play.

#### Self-perceived confidence and awareness toward teledentistry

All participants were requested to rate their perceptions of teledentistry before and after participating in the gamified online role-play (Supplementary material 1). The self-perceived assessment was developed based on previous literature^39–42^. The assessment scores would inform whether or not the participants could improve their self-perceived confidence and awareness through a learning activity. The assessment consisted of two parts, which were (1) self-perceived confidence and (2) self-perceived awareness. Each part contained six items, which were similar between the pre- and post-assessments. All items were designed using a 5-point Likert scale, where 1 being ‘strongly disagree’ and 5 being ‘strongly agree’.

#### Satisfactions toward the gamified online role-play

All participants were asked to complete the satisfaction questionnaire after participating in the gamified online role-play, to investigate whether or not they felt satisfied with their learning (Supplementary material 2). The questionnaire was developed based on previous literature regarding gamification and role-play^41–44^. Most of the items were designed using a 5-point Likert scale, where 1 being ‘very dissatisfied’ and 5 being ‘very satisfied’. They were grouped into three aspects, which were (1) Perceived usefulness, (2) Perceived ease of use, and (3) Perceived enjoyment.

#### Learner experiences within the gamified online role-play

Semi-structured interviews were conducted with the purposively selected participants to gather in-depth information regarding their learning experiences within the gamified online role-play. This technique allowed researchers to ask additional interesting topics raised from the responses of participants. A topic guide for interviews were constructed based on the findings of previous literature^45–47^. The interview was conducted in a private room by a researcher who was trained in conducting qualitative research including interviews. The interview sessions took approximately 45–60 minutes, where all responses from participants were recorded using a digital audio recorder with their permission. The recorded audios were transcribed using a verbatim technique by a transcription service under a confidential agreement.

#### Validity and reliability of data collection tools

To enhance the quality of self-perceived assessment and satisfaction questionnaire, they were piloted and revised to assure their validity and reliability. According to the content validity, three experts in advanced general dentistry were asked to evaluate the questionnaire, where problematic items were iteratively revised until they achieved the index of item-objective congruence (IOC) higher than 0.5. To perform a test–retest reliability, the validated versions of both self-perceived assessment and satisfaction questionnaire were afterwards piloted in residents from other programs, and the data were analyzed using an intraclass correlation coefficient (ICC), where the values of all items were 0.7 or greater. The data from the first pilot completion of both data collection tools were analyzed using Cronbach’s alpha to ensure the internal consistency of all constructs. The problematic items were deleted to achieve the coefficient alpha of 0.7 or greater for all constructs, which was considered as acceptable internal consistency.

### Data analysis

The quantitative data retrieved from self-perceived assessment and satisfaction questionnaire were analyzed with the Statistical Package for Social Sciences software (SPSS, version 29, IBM Corp.). Descriptive statistics were performed to present an overview of the data. The scores from pre- and post-assessments were analyzed using a paired sample t-test to evaluate whether or not the participants would better self-perceive their confidence and awareness in teledentistry after participating in the gamified online role-play. One-way analysis of variance (ANOVA) was conducted to compare whether or not there were statistically significant differences in self-perceived assessment and satisfaction scores among the three academic years.

The qualitative data retrieved from semi-structured interviews were analyzed using a framework analysis, where its procedure involved transcription, familiarization with the interview data, coding, developing an analytical framework, indexing, charting, and data interpreting qualitative findings^48^. In this research, the initial codes had been pre-defined from previous literature and subsequently adjusted following the analysis of each transcript to develop an analytical framework (themes and subthemes), requiring several iterations until no additional codes emerged. Subsequently, the established categories and codes were applied consistently across all transcripts (indexing). The data from each transcript were then charted to develop a matrix, facilitating the management and summarization of qualitative findings. This method enabled the researchers to compare and contrast differences within the data and to identify connections between categories, thereby exploring their relationships and informing data interpretation.

The procedure of framework analysis necessitated a transparent process for data management and interpretation of emerging themes to ensure the robustness of research^49^. The transparency of this analytic approach enabled two researchers (C.Te. and K.S.) to independently analyze the qualitative data, and the emerging themes afterwards were discussed to obtain consensus among the researchers. This technique can be considered as a triangular approach to assure the intercoder reliability and internal validity of this research. The transparent process also allowed an external expert in dental education to verify the accuracy of the analysis. All emerging themes and the decision on data saturation were based on a discussion of all researchers until an agreement was made. NVivo (version 14, QSR International) was used to performed the qualitative data analysis. Subsequently, a conceptual framework was constructed to demonstrate emerging themes and subthemes together with their relationships.

### Ethical consideration

The ethical approval for the study was approved by the Institutional Review Board of Faculty of Dentistry and Faculty of Pharmacy, Mahidol University on 29^th^ September 2022, the ethical approval number: MU-DT/PY-IRB 2022/049.2909. All methods were performed in accordance with the relevant guidelines and regulations. Although the data were not anonymous in nature as they contained identifiable data, they were coded prior to the analysis to assure confidentiality of participants.

### Informed consent

Informed consent was obtained from all participants.

## Results

### Research participants

There were 18 residents from Year 1 to 3 of the Residency Training Program in Advanced General Dentistry who participated in this research (six from each year). Of these, there were 14 females and 4 males. There was no participant dropout, as all of them completed all required tasks, including the pre- and post-perceived assessments, gamified online role-play, and satisfaction questionnaire. According to the purposive sampling, the participants from the quantitative phase were selected for semi-structured interviews by considering sex, year of study, and self-perceived assessment scores. Twelve students (ten females and two males) participated in semi-structured interviews, where their characteristics are presented in Table 1.

**Table 1:** Participants who participated in semi-structured interviews.

Participant	Sex	Age	Year of study	Improvement in self-perceived confidence	Improvement in self-perceived awareness
1	Female	29	3	2.0	0.6
2	Female	28	1	1.5	0.9
3	Female	28	2	1.0	0.9
4	Male	29	3	0.7	1.0
5	Female	28	2	1.5	0
6	Female	29	2	0.5	0.7
7	Female	27	1	1.2	0.1
8	Female	28	2	0.2	0.6
9	Female	29	3	0.5	0.1
10	Female	28	1	0.6	0
11	Male	29	2	0	0
12	Female	29	1	0	0

### Internal consistency of all constructs

The data collected from the research participants, in addition to the pilot samples, were analyzed with Cronbach’s alpha to confirm the internal consistency. The coefficient alpha of all constructs demonstrated high internal consistency, as demonstrated in Table 2.

**Table 2:** Cronbach’s alpha of each construct.

Constructs	Coefficient alpha
Self-perceived confidence (6 items)	0.90
Self-perceived awareness (6 items)	0.78
Self-perceived usefulness (6 items)	0.81
Self-perceived ease of use (6 items)	0.79
Self-perceived enjoyment (6 items)	0.91

### Self-perceived assessments toward confidence and awareness of teledentistry

There were statistically significant increases in the assessment scores of self-perceived confidence and awareness after participating in the gamified online role-play (*P* < 0.001). According to Table 3, there was an increase in self-perceived confidence from 3.38 (SD = 0.68) for the pre-assessment to 4.22 (SD = 0.59) for the post-assessment (*P* < 0.001). The findings of self-perceived awareness also showed score improvement from 4.16 (SD = 0.48) to 4.55 (SD = 0.38) after interacting with the simulated patient (*P* < 0.001).

**Table 3:** Self-perceived assessments toward confidence and awareness of teledentistry.

Self-perceived assessments	Pre-assessment score Mean (SD)	Post-assessment score Mean (SD)	*P-*value
Self-perceived confidence	3.38 (0.68)	4.22 (0.59)	< 0.001
Self-perceived awareness	4.16 (0.48)	4.55 (0.38)	< 0.001

Self-perceived assessments on a 5-point Likert scale, ranging from ‘Strongly disagree’ (1) to ‘Strongly agree’ (5).

The significance level was taken at *P* < 0.05.

**Figure 2:**
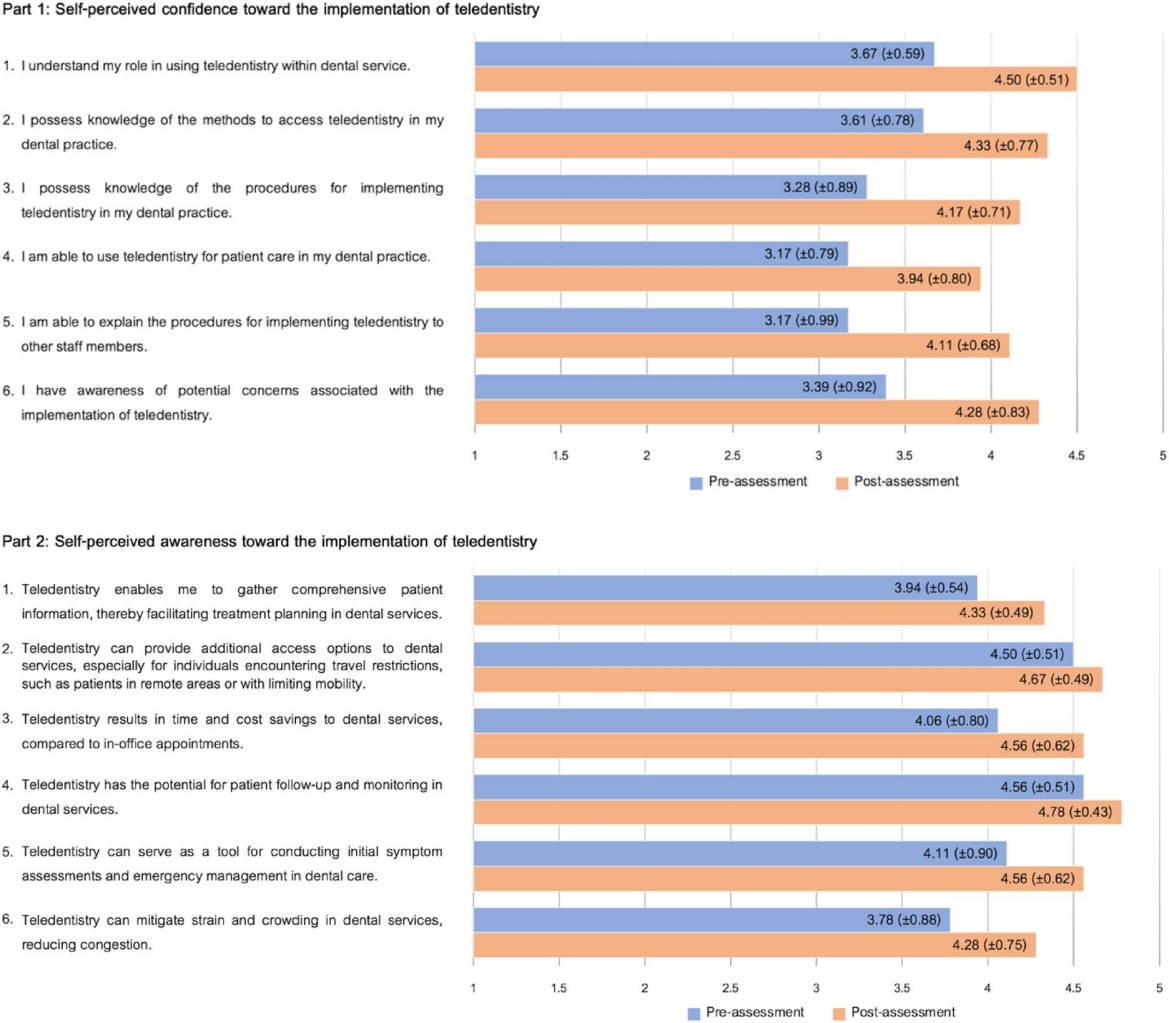
Self-perceived assessments toward confidence and awareness of teledentistry.

According to Fig. 2, participants demonstrated a higher level of self-perceived assessments for both self-confidence and awareness in all aspects after participating in the gamified online role-play for teledentistry training.

When comparing the self-perceived assessment scores toward confidence and awareness in the use of teledentistry among the three years of study (Year 1–3), there were no statistically significant differences in the pre-assessment, post-assessment score, and score difference (Table 4).

**Table 4:** Pre- and post-assessment scores among three groups.

Self-perceived assessments	Year 1 Mean (SD)	Year 2 Mean (SD)	Year 3 Mean (SD)	*P*-value
Self-perceived confidence				
Pre-assessment score	3.06 (0.17)	3.83 (0.80)	3.25 (0.71)	0.115
Post-assessment score	3.97 (0.82)	4.44 (0.46)	4.28 (0.52)	0.346
Score improvement	0.89 (0.67)	0.61 (0.55)	1.03 (0.56)	0.484
Self-perceived awareness				
Pre-assessment score	4.13 (0.44)	4.36 (0.47)	3.97 (0.54)	0.399
Post-assessment score	4.44 (0.43)	4.75 (0.17)	4.44 (0.44)	0.787
Score improvement	0.31 (0.36)	0.39 (0.37)	0.47 (0.34)	0.728

Self-perceived assessments on a 5-point Likert scale, ranging from ‘Strongly disagree’ (1) to ‘Strongly agree’ (5).

The significance level was taken at *P* < 0.05.

### Satisfactions toward the use of gamified online role-play

**Figure 3:**
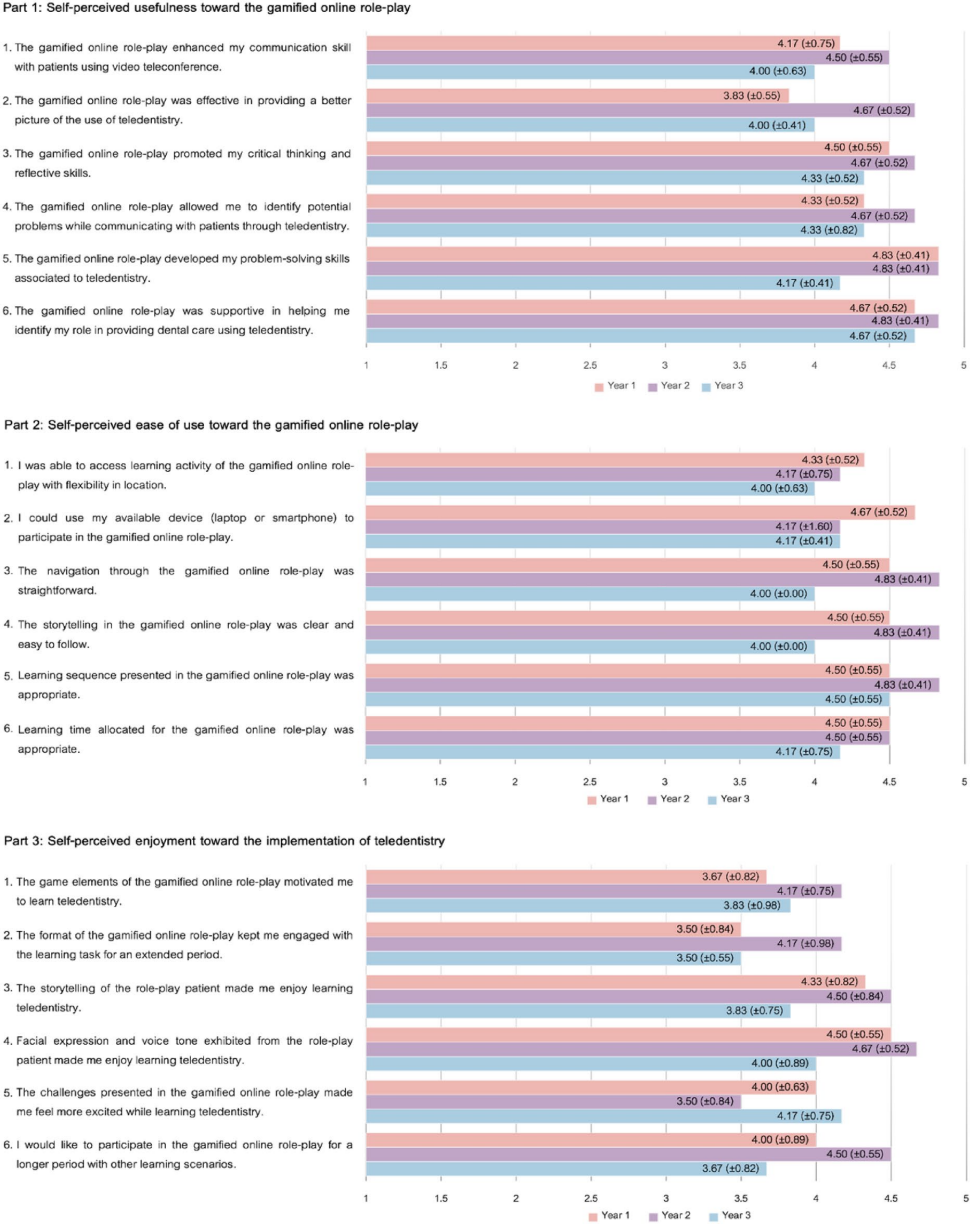
Satisfactions toward the use of gamified online role-play.

According to Fig. 3, participants exhibited high levels of satisfaction with the use of gamified online role-play across all three aspects. The aspect of usefulness received the highest satisfaction rating with a score of 4.44 (SD = 0.23) out of 5, followed by ease of use and enjoyment, scoring 4.40 (SD = 0.23) and 4.03 (SD = 0.21), respectively. Particularly, participants expressed the highest satisfaction levels regarding the usefulness of gamified online role-play for identifying their role (Mean = 4.72, SD = 0.46) and developing problem-solving skills associated with teledentistry (Mean = 4.61, SD = 0.50). Additionally, they reported satisfaction with the learning sequence presented in the gamified online role-play (Mean = 4.61, SD = 0.50). However, participants did not strongly perceive that the format of the gamified online role-play could engage them with the learning task for an extended period (Mean = 3.72, SD = 0.83).

When comparing the satisfaction levels perceived by participants from different academic years (Table 5), no statistically significant differences were observed among the three groups for all three aspects (*P* > 0.05).

**Table 5:** Satisfactions toward the use of gamified online role-play.

Satisfactions	Year 1 Mean (SD)	Year 2 Mean (SD)	Year 3 Mean (SD)	*P*-value
Self-perceived usefulness	4.39 (0.46)	4.69 (0.47)	4.25 (0.66)	0.158
Self-perceived ease of use	4.50 (0.29)	4.56 (0.51)	4.14 (0.63)	0.263
Self-perceived enjoyment	4.00 (0.39)	4.25 (0.25)	3.83 (0.72)	0.570
Overall	4.30 (0.26)	4.50 (0.23)	4.07 (0.22)	0.997

User satisfactions on a 5-point Likert scale, ranging from ‘Strongly disagree’ (1) to ‘Strongly agree’ (5).

The significance level was taken at *P* < 0.05.

### Learner experiences within the gamified online role-play

Following the framework analysis of qualitative data, there were five emerging themes, including: (1) learner profile, (2) learning settings of the gamified online role-play, (3) pedagogical components, (4) interactive functions, and (5) educational impact.

### Theme 1: Learner profile

Learner experience and preferences appeared to have impact on how the participants perceived the use of gamified online role-play for teledentistry training. When learners preferred role-play or realized benefits of teledentistry, they were likely to support this learning intervention. In addition, they could have seen an overall picture of the assigned tasks before participating in this research.

*“I had experience with a role-play activity when I was dental undergraduates, and I like this kind of learning where someone role-plays a patient with specific personalities in various contexts. This could be a reason why I felt interested to participate in this task (the gamified online role-play). I also believed that it would be supportive for my clinical practice.”*

*Participant 12, Year 1, Female*

*“Actually, I' have seen in several videos (about teledentistry), where dentists were teaching patients to perform self-examinations, such as checking their own mouth and taking pictures for consultations. Therefore, I could have thought about what I would experience during the activity (within the gamified online role-play).”*

*Participant 8, Year 2, Female*

### Theme 2: Learning settings of the gamified online role-play

#### Subtheme 2.1: Location

Participants had agreed that the location for conducting a gamified online role-play should be in a private room without any disturbances, enabling learners to focus on the simulated patient. This could allow them to effectively communicate and understand of the needs of patient, leading to a better grasp of lesson content. In addition, the environments of both learners and simulated patient should be authentic to the learning quality.

*“The room should be a private space without any disturbances. This will make us feel confident and engage in conversations with the simulated patient.”*

*Participant 10, Year 1, Female*

*“… simulating a realistic environment can engage me to interact with the simulated patient more effectively ...”*

*Participant 8, Year 2, Female*

#### Subtheme 2.2: Time allocated for the gamified online role-play

The time allocated for the gamified online role-play in this research was considered as appropriate, as participants believed that a 30-minutes period should be suitable to take information and afterwards give some advice to their patient. In addition, a 10-minutes discussion on how they interact with the patient could be supportive for participants to enhance their competencies in the use of teledentistry.

*“… it would probably take about 20 minutes because we would need to gather a lot of information … it might need some time to request and gather various information … maybe another 10-15 minutes to provide some advice.”*

*Participant 7, Year 1, Female*

*“I think during the class … we could allocate around 30 minutes for role-play, … we may have discussion of learner performance for 10-15 minutes ... I think it should not be longer than 45 minutes in total.”*

*Participant 6, Year 2, Female*

#### Subtheme 2.3: Learning consequence within a postgraduate curriculum

Most participants suggested that the gamified online role-play in teledentistry should be arranged in the first year of their postgraduate program. This could maximize the effectiveness of online role-play, as they would be able to implement teledentistry for their clinical practice since the beginning of their training. However, some participants suggested that this learning approach could be rearranged in either second or third year of the program. As they already had experience in clinical practice, the gamified online role-play would reinforce their competence in teledentistry.

*"Actually, it would be great if this session could be scheduled in the first year … I would feel more comfortable when dealing with my patients through an online platform."*

*Participant 11, Year 2, Male*

*"I believe this approach should be implemented in the first year because it allows students to be trained in teledentistry before being exposed to real patients. However, if this approach is implemented in either the second or third year when they have already had experience in patient care, they would be able to better learn from conversations with simulated patients."*

*Participant 4, Year 3, Male*

### Theme 3: Pedagogical components

#### Subtheme 3.1: Learning content

Learning content appeared to be an important component of pedagogical aspect, as it would inform what participants should learn from the gamified online role-play. Based on the interview data, participants reported they could learn how to use a video teleconference platform for teledentistry. The conditions of simulated patient embedded in an online role-play also allowed them to realize the advantages of teledentistry. In addition, dental problems assigned to the simulated patient could reveal the limitations of teledentistry for participants.

*“The learning tasks (within the gamified online role-play) let me know how to manage patients through the teleconference.”*

*Participant 5, Year 2, Female*

*“… there seemed to be limitations (of teledentistry) … there could be a risk of misdiagnosis … the poor quality of video may lead to diagnostic errors … it is difficult for patients to capture their oral lesions.”*

*Participant 3, Year 2, Female*

#### Subtheme 3.2: Feedback

During the use of online role-play, the simulated patient can provide formative feedback to participants through facial expressions and tones of voice, enabling participants to observe and learn to adjust their inquiries more accurately. In addition, at the completion of the gamified online role-play, summative feedback provided by instructors could summarize the performance of participants leading to further improvements in the implementation of teledentistry.

*“I knew (whether or not I interacted correctly) from the gestures and emotions of the simulated patient between the conversation. I could have learnt from feedback provided during the role-play, especially from the facial expressions of the patient.”*

*Participant 11, Year 2, Male*

*“The feedback provided at the end let me know how well I performed within the learning tasks.”*

*Participant 2, Year 1, Female*

### Theme 4: Interactive functions

#### Subtheme 4.1: The authenticity of the simulated patient

Most participants believed that a simulated patient with high acting performance could enhance the flow of role-play, allowing learners to experience real consequences. The appropriate level of authenticity could engage learners with the learning activity, as they would have less awareness of time passing in the state of flow. Therefore, they could learn better from the gamified online role-play.

*"It was so realistic. ... This allowed me to talk with the simulated patient naturally ... At first, when we were talking, I was not sure how I should perform … but afterwards I no longer had any doubts and felt like I wanted to explain things to her even more."*

*Participant 3, Year 2, Female*

*"At first, I believed that if there was a factor that could influence learning, it would probably be a simulated patient. I was impressed by how this simulated patient could perform very well. It made the conversation flow smoothly and gradually."*

*Participant 9, Year 3, Female*

#### Subtheme 4.2: Entertaining features

Participants were likely to be satisfied with the entertaining features embedded in the gamified online role-play. They felt excited when they were being exposed to the unrevealed challenge which they had randomly selected. In addition, participants suggested to have more learning scenarios or simulated patients where they could randomly select to enhance randomness and excitement.

*“It was a playful experience while communicating with the simulated patient. There are elements of surprise from the challenge cards that make the conversation more engaging, and I did not feel bored during the role-play.”*

*Participant 4, Year 3, Male*

*“I like the challenge card we randomly selected, as we had no idea what we would encounter … more scenarios like eight choices and we can randomly choose to be more excited. I think we do not need additional challenge cards, as some of them have already been embedded in patient conditions.”*

*Participant 5, Year 2, Female*

#### Subtheme 4.3: Level of difficulty

Participants suggested the gamified online role-play to have various levels of difficulty, so learners could have a chance to select a suitable level for their competence. The difficulties could be represented through patient conditions (e.g., systemic diseases or socioeconomic status), personal health literacy, and emotional tendencies. They also recommended to design the gamified online role-play to have different levels where learners could select an option that is suitable for them.

*“The patient had hidden their information, and I needed to bring them out from the conversation.”*

*Participant 12, Year 1, Female*

*“Patients' emotions could be more sensitive to increase level of challenges. This can provide us with more opportunities to enhance our management skills in handling patient emotions.”*

*Participant 11, Year 2, Male*

*“… we can gradually increase the difficult level, similar to playing a game. These challenges could be related to the simulated patient, such as limited knowledge or difficulties in communication, which is likely to occur in our profession.”*

*Participant 6, Year 2, Female*

### Theme 5: Educational impact

#### Subtheme 5.1: Self-perceived confidence in teledentistry

##### Communication skills

Participants were likely to perceive that they could learn from the gamified online role-play and felt more confident in the use of teledentistry. This educational impact was mostly achieved from the online conversation within the role-play activity, where the participants could improve their communication skills through a video teleconference platform.

*“I feel like the online role-play was a unique form of learning. I believe that I gained confidence from the online communication the simulated patient. I could develop skills to communicate effectively with real patients.”*

*Participant 11, Year 2, Male*

*“I believe it support us to train communication skills ... It allowed us to practice both listening and speaking skills more comprehensively.”*

*Participant 4, Year 3, Male*

##### Critical thinking and problem-solving skills

In addition to communication skills, participants reported that challenges embedded in the role-play allowed them to enhance critical thinking and problem-solving skills, which were a set of skills required to deal with potential problems in the use of teledentistry.

*"It was a way of training before experiencing real situations … It allowed us to think critically whether or not what we performed with the simulated patients was appropriate."*

*Participant 7, Year 1, Female*

*“It allowed us to learn how to effectively solve the arranged problems in simulated situation. We needed to solve problems in order to gather required information from the patient and think about how to deliver dental advice through teledentistry.”*

*Participant 11, Year 2, Male*

#### Subtheme 5.2: Self perceived awareness in teledentistry

Participants believed that they could realize the necessity of teledentistry from the gamified online role-play. The storytelling or patient conditions allowed learners to understand how teledentistry could have both physical and psychological support for dental patients.

*“From the activity, I would consider teledentistry as a convenient tool for communicating with patients, especially if a patient cannot go to a dental office”.*

*Participant 5, Year 2, Female*

*“I learned about the benefits of teledentistry, particularly in terms of follow-up. The video conference platform could support information sharing, such as drawing images or presenting treatment plans, to patients.”*

*Participant 8, Year 2, Female*

### A conceptual framework of learning experience within a gamified online role-play

**Figure 4:**
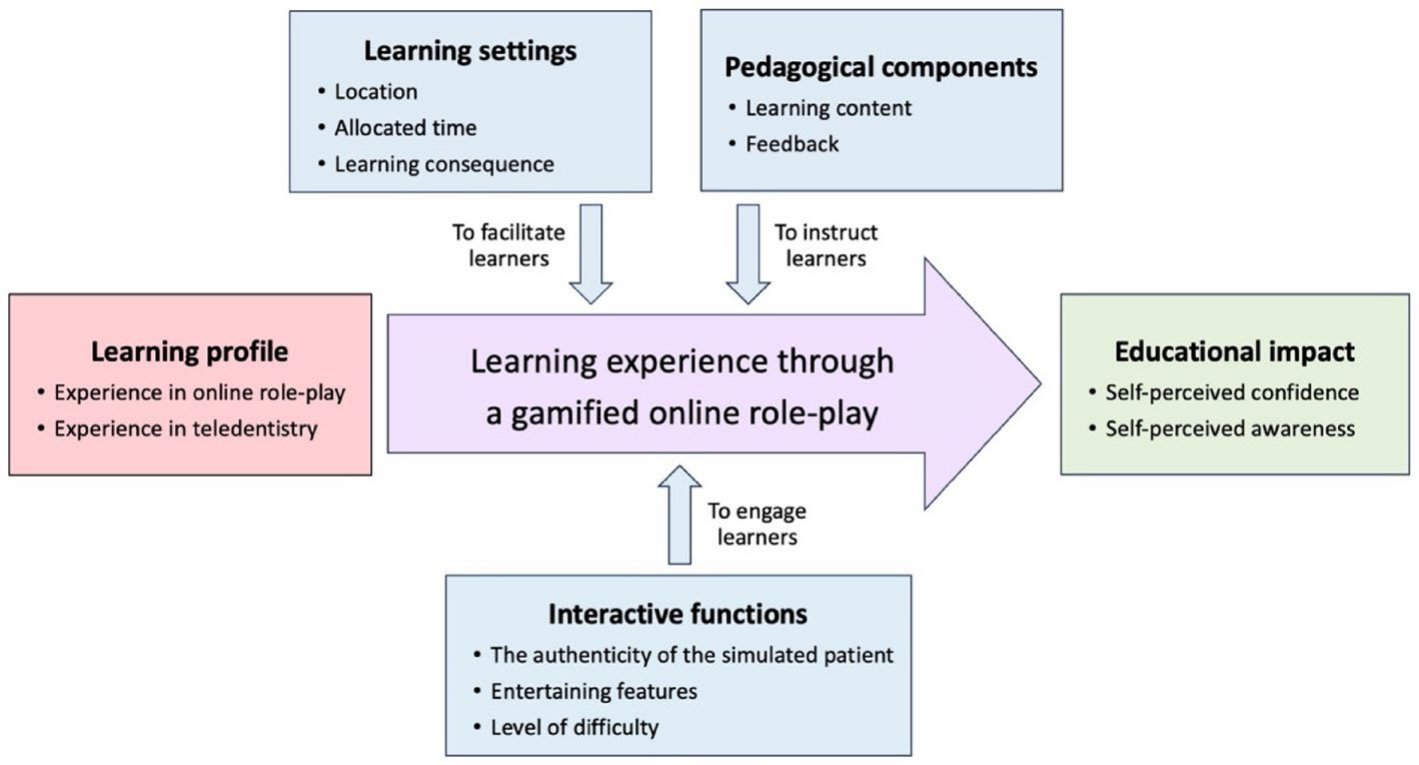
The conceptual framework of key elements in designing a gamified online role-play.

Based on the qualitative findings, a conceptual framework was developed in which a gamified online role-play was conceptualized as a learning strategy in supporting learners to be able to implement teledentistry in their clinical practice (Fig. 4).

The conceptual framework has revealed key elements to be considered in designing a gamified online role-play. Learner profile, learning settings, pedagogical components, and interactive functions are considered as influential factors toward user experience within the gamified online role-play. The well-designed learning activity will support learners to achieve expected learning outcomes, considered as educational impact of the gamified online role-play. The contributions of these five key elements to the design of gamified online role-play were interpreted, as follows:

**Learner profile:** This element tailors the design of gamified online role-plays for teledentistry training involves considering the background knowledge, skills, and experiences of target learners to ensure relevance and engagement.

**Learning settings:** The element focuses the planning for gamified online role-plays in teledentistry training involves selecting appropriate contexts, such as location and timing, to enhance accessibility and achieve learning outcomes effectively.

**Pedagogical components:** This element emphasizes the alignment between learning components and learning outcomes within gamified online role-plays, to ensure that the content together with effective feedback design can support learners in improving their competencies from their mistakes.

**Interactive functions:** This element highlights interactivity features integrated into gamified online role-plays, such as the authenticity and entertaining components to enhance immersion and engagement, together with game difficulty for optimal flow. All these features should engage learners with the learning activities until the achievement of learner outcomes.

**Educational impact:** This element represents the expected learning outcomes, which will inform the design of learning content and activities within gamified online role-plays. In addition, this element could be considered to evaluate the efficacy of gamified online role-plays, reflecting how well learning designs align with the learning outcomes.

## Discussion

A gamified online role-play can be considered as a learning strategy for teledentistry according to its educational impact. This pedagogical approach could mimic real-life practice, where dental learners could gain experience in the use of teledentistry in simulated situations before interacting with actual patients. Role-play could provide learners opportunities to develop their required competencies, especially communication and real-time decision-making skills, in a predictable and safe learning environment^20,23,46^. Potential obstacles could also be arranged for learners to deal with, leading to the enhancement of problem-solving skill^50^. In addition, the recognition of teledentistry benefits can enhance awareness and encourage its adoption and implementation, which could be explained by the technology acceptance model^51^. Therefore, a gamified online role-play with a robust design and implementation appeared to have potential in enhancing self-perceived confidence and awareness in the use of teledentistry.

The pedagogical components comprised learning content, which was complemented by assessment and feedback. Learners could develop their competence with engagement through the learning content, gamified by storytelling of the online role-play^52,53^. Immediate feedback provided through facial expression and voice tone of simulated patients allowed participants to learn from their failure, considered as a key feature of game-based learning^29,45^. The discussion of summative feedback provided from an instructor at the end of role-play activity could support a debriefing process enabling participants to reflect their learning experience, considered as important of simulation-based game^54^. These key considerations should be initially considered in the design of gamified online role-play.

The interactive functions can be considered as another key component for designing and evaluating the gamified online role-play^45^. Several participants enjoyed with a learning process within the gamified online role-play and suggested it to have more learning scenarios. In other words, this tool could engage learners with an instructional process, leading to the achievement of learning outcomes^29,45^. As challenge and randomness appear to be game elements^32,33^, this learning intervention assigned a set of cards with obstacle tasks for learners to randomly pick up before interacting with simulated patients, which was perceived by participants as a feature to make the role-play more challenging and engaging. This is consistent with previous research, where challenging content for simulated patients could make learners more engaged with a learning process^55^. However, the balance between task challenges and learner competencies is certainly required for the design of learning activities^56,57^. The authenticity of simulated patient and immediate feedback could also affect the game flow, leading to the enhancement of learner engagement^45^. These elements could engage participants with a learning process, leading to the enhancement of educational impact.

The educational settings for implementing gamified online role-play into dental curriculum should be another concern. This aspect has been recognized as significant in existing evidence^45^. As this research found no significant differences in all aspects among the three groups of learners, this learning intervention demonstrated the potential for its implementation at any time of postgraduate dental curriculum. This argument can be supported by previous evidence where a role-play could be adaptable for learning at any time, as it requires a short learning period but provides learners with valuable experience prior to being exposed in real-life scenarios^58^. This strategy also provides opportunities for learners who have any question or concern to seek advice or guidance from their instructors^59^. Although the gamified online role-play can be arranged in the program at any time, the first academic year should be considered, as dental learners would be confidence in implementing teledentistry for their clinical practice.

While a gamified online role-play demonstrated its strengths as an interactive learning strategy specifically for teledentistry, there are a couple of potential drawbacks that need to be addressed. The requirement for synchronous participation could limit the flexibility of access time for learners (synchronous interactivity limitation). With only one learner able to engage with a simulated patient at a time (limited participants), more simulated patients would be required if there are a number of learners, otherwise they would need to wait for their turn. Time and resources are significantly required for preparing simulated patients^60^. Despite the use of trained and calibrated professional actors/actresses, inauthenticity may be perceived during role-plays, requiring a significant amount of effort to achieve both interactional and clinical authenticities^46^. Future research could investigate asynchronous learning approaches utilizing non-player character (NPC) controlled by an artificial intelligence system as a simulated patient. This setup would enable multiple learners to have the flexibility to engage with the material at their own pace and at times convenient to them^29^. While there are potential concerns about using gamified online role-plays, this interactive learning intervention offers opportunities for dental professionals to enhance their teledentistry competency in a safe and engaging environment.

Albeit the robust design and data collection tools to assure reliability and validity as well as transparency of this study, a few limitations were raised leading to a potential of further research. While this research recruited only postgraduate students to evaluate the feasibility of gamified online role-play in teledentistry training, further research should include not only experienced dental practitioners but also undergraduate students to confirm its potential use in participants with different learner profiles. More learning scenarios in other dental specialties should also be included to validate its effectiveness, as different specialties could have different limitations and variations. Additional learning scenarios from various dental disciplines should be considered to validate the effectiveness of gamified online role-plays, as different specialties may present unique limitations and variations. A randomized controlled trial with robust design should be required to compare the effectiveness of gamified online role-play with different approaches in training the use of teledentistry.

## Conclusions

This research supports the design and implementation of a gamified online role-play in dental education, as dental learners could develop self-perceived confidence and awareness with satisfaction. A well-designed gamified online role-play is necessary to support learners to achieve expected learning outcomes, and the conceptual framework developed in this research can serve as a guidance to design and implement this interactive learning strategy in dental education. However, further research with robust design should be required to validate and ensure the educational impact of gamified online role-play in dental education. Additionally, efforts should be made to develop gamified online role-play in asynchronous learning approaches to enhance the flexibility of learning activities.

### Supplementary Information

Supplementary Information 1.

Supplementary Information 2.

## Data Availability

The data that support the findings of this study are available from the corresponding author, up-on reasonable request. The data are not publicly available due to information that could compromise the privacy of research participants.

## References

[1] Van Dyk L (2014). A review of telehealth service implementation frameworks. Int. J. Environ. Res. Public Health.

[2] Bartz CC (2016). Nursing care in telemedicine and telehealth across the world. Soins..

[3] Lin, G.S.S., Koh, S.H., Ter, K.Z., Lim, C.W., Sultana, S., Tan, W.W. Awareness, knowledge, attitude, and practice of teledentistry among dental practitioners during COVID-19: A systematic review and meta-analysis. *Medicina (Kaunas).* **58**(1), 130 (2022). 10.3390/medicina58010130 PMC8781277 35056438

[4] Wolf, T.G., Schulze, R.K.W., Ramos-Gomez, F., Campus, G. Effectiveness of telemedicine and teledentistry after the COVID-19 pandemic. *Int. J. Environ. Res. Public Health.* **19**(21), 13857 (2022). 10.3390/ijerph192113857 PMC9656303 36360734

[5] Gajarawala SN, Pelkowski JN (2021). Telehealth benefits and barriers. J. Nurse Pract..

[6] Jampani ND, Nutalapati R, Dontula BS, Boyapati R (2011). Applications of teledentistry: A literature review and update. J. Int. Soc. Prev. Community Dent..

[7] Khan SA, Omar H (2013). Teledentistry in practice: literature review. Telemed. J. E. Health..

[8] Baheti MJBS, Toshniwal NG, Misal A (2014). Teledentistry: A need of the era. Int. J. Dent. Med. Res..

[9] Datta N, Derenne J, Sanders M, Lock JD (2020). Telehealth transition in a comprehensive care unit for eating disorders: Challenges and long-term benefits. Int. J. Eat. Disord..

[10] Bursell SE, Brazionis L, Jenkins A (2012). Telemedicine and ocular health in diabetes mellitus. Clin. Exp. Optom..

[11] da Costa CB, Peralta FDS, Ferreira de Mello ALS (2020). How has teledentistry been applied in public dental health services? An integrative review. Telemed. J. E. Health..

[12] Heckemann B, Wolf A, Ali L, Sonntag SM, Ekman I (2016). Discovering untapped relationship potential with patients in telehealth: A qualitative interview study. BMJ Open..

[13] Pérez-Noboa B, Soledispa-Carrasco A, Padilla VS, Velasquez W (2021). Teleconsultation apps in the COVID-19 pandemic: The case of Guayaquil City, Ecuador. IEEE Eng. Manag. Rev..

[14] Wamsley CE, Kramer A, Kenkel JM, Amirlak B (2020). Trends and challenges of telehealth in an academic institution: The unforeseen benefits of the COVID-19 global pandemic. Aesthetic Surg. J..

[15] Jonasdottir SK, Thordardottir I, Jonsdottir T (2022). Health professionals’ perspective towards challenges and opportunities of telehealth service provision: A scoping review. Int. J. Med. Inform..

[16] Tan SHX, Lee CKJ, Yong CW, Ding YY (2021). Scoping review: Facilitators and barriers in the adoption of teledentistry among older adults. Gerodontology..

[17] Minervini G, Russo D, Herford AS (2022). Teledentistry in the management of patients with dental and temporomandibular disorders. BioMed. Res. Int..

[18] Edirippulige S, Armfield N (2017). Education and training to support the use of clinical telehealth: A review of the literature. J. Telemed. Telecare..

[19] Mariño R, Ghanim A (2013). Teledentistry: A systematic review of the literature. J. Telemed. Telecare..

[20] Armitage-Chan E, Whiting M (2016). Teaching professionalism: Using role-play simulations to generate professionalism learning outcomes. J. Vet. Med. Educ..

[21] Spyropoulos F, Trichakis I, Vozinaki A-E (2022). A narrative-driven role-playing game for raising flood awareness. Sustainability..

[22] Jiang WK, Wang DY, Liu GC (2020). Role-play in endodontic teaching: A case study. Chin. J. Dent. Res..

[23] Vizeshfar F, Zare M, Keshtkaran Z (2019). Role-play versus lecture methods in community health volunteers. Nurse Educ. Today..

[24] Nestel D, Tierney T (2007). Role-play for medical students learning about communication: Guidelines for maximising benefits. BMC Med. Educ..

[25] Gelis A, Cervello S, Rey R (2020). Peer role-play for training communication skills in medical students: A systematic review. Simulat. Health..

[26] Cornelius S, Gordon C, Harris M (2011). Role engagement and anonymity in synchronous online role play. Int. Rev. Res. Open Distrib. Learn..

[27] Bell M (2001). Online role-play: Anonymity, engagement and risk. Educ. Med. Int..

[28] Sipiyaruk K, Gallagher JE, Hatzipanagos S, Reynolds PA (2018). A rapid review of serious games: From healthcare education to dental education. Eur. J. Dent. Educ..

[29] Sipiyaruk K, Hatzipanagos S, Reynolds PA, Gallagher JE (2021). Serious games and the COVID-19 pandemic in dental education: An integrative review of the literature. Computers..

[30] Morse, J.M., Niehaus, L. *Mixed Method Design: Principles and Procedures.* (2016).

[31] Creswell JW (2009). Research Design: Qualitative, Quantitative, and Mixed Methods Approaches.

[32] Cheng VWS, Davenport T, Johnson D, Vella K, Hickie IB (2019). Gamification in apps and technologies for improving mental health and well-being: Systematic review. JMIR Ment. Health..

[33] Gallego-Durán FJ, Villagrá-Arnedo CJ, Satorre-Cuerda R, Compañ-Rosique P, Molina-Carmona R, Llorens-Largo F (2019). A guide for game-design-based gamification. Informatics..

[34] Gee JP, Salen K (2008). Learning and games. The Ecology of Games: Connecting Youth, Games, and Learning.

[35] Cheung KL, ten Klooster PM, Smit C, de Vries H, Pieterse ME (2017). The impact of non-response bias due to sampling in public health studies: A comparison of voluntary versus mandatory recruitment in a Dutch national survey on adolescent health. BMC Public Health..

[36] Murairwa S (2015). Voluntary sampling design. Int. J. Adv. Res. Manag. Social Sci..

[37] Chow S-C, Shao J, Wang H, Lokhnygina Y (2017). Sample Size Calculations in Clinical Research.

[38] Palinkas LA, Horwitz SM, Green CA, Wisdom JP, Duan N, Hoagwood K (2015). Purposeful sampling for qualitative data collection and analysis in mixed method implementation research. Administration Policy Mental Health Mental Health Services Res..

[39] McIlvried DE, Prucka SK, Herbst M, Barger C, Robin NH (2008). The use of role-play to enhance medical student understanding of genetic counseling. Genet. Med..

[40] Schlegel C, Woermann U, Shaha M, Rethans J-J, van der Vleuten C (2012). Effects of communication training on real practice performance: A role-play module versus a standardized patient module. J. Nursing Educ..

[41] Manzoor IMF, Hashmi NR (2012). Medical students’ perspective about role-plays as a teaching strategy in community medicine. J. Coll. Physicians Surg. Pak..

[42] Cornes S, Gelfand JM, Calton B (2021). Foundational telemedicine workshop for first-year medical students developed during a pandemic. MedEdPORTAL..

[43] King J, Hill K, Gleason A (2015). All the world’sa stage: Evaluating psychiatry role-play based learning for medical students. Austral. Psychiatry..

[44] Arayapisit T, Pojmonpiti D, Dansirisomboon K, Jitverananrangsri K, Poosontipong D, Sipiyaruk K (2023). An educational board game for learning orofacial spaces: An experimental study comparing collaborative and competitive approaches. Anatomical Sci. Educ..

[45] Sipiyaruk, K., Hatzipanagos, S., Vichayanrat, T., Reynolds, P.A., Gallagher, J.E. Evaluating a dental public health game across two learning contexts. *Educ. Sci.* **12**(8), 517 (2022).

[46] Pilnick A, Trusson D, Beeke S, O’Brien R, Goldberg S, Harwood RH (2018). Using conversation analysis to inform role play and simulated interaction in communications skills training for healthcare professionals: Identifying avenues for further development through a scoping review. BMC Med. Educ..

[47] Lane C, Rollnick S (2007). The use of simulated patients and role-play in communication skills training: A review of the literature to August 2005. Patient Educ. Counseling..

[48] Gale NK, Heath G, Cameron E, Rashid S, Redwood S (2013). Using the framework method for the analysis of qualitative data in multi-disciplinary health research. BMC Med. Res. Methodol..

[49] Ritchie J, Lewis J, Nicholls CM, Ormston R (2014). Qualitative Research Practice: A Guide for Social Science Students and Researchers.

[50] Chen JC, Martin AR (2015). Role-play simulations as a transformative methodology in environmental education. J. Transform. Educ..

[51] Davis, F. D. Perceived usefulness, perceived ease of use, and user acceptance of information technology. *Manag. Inform. Syst. Quart.* **13**(3), 319–340 (1989).

[52] Novak E, Johnson TE, Tenenbaum G, Shute VJ (2016). Effects of an instructional gaming characteristic on learning effectiveness, efficiency, and engagement: Using a storyline for teaching basic statistical skills. Interact. Learn. Environ..

[53] Marchiori EJ, Torrente J, del Blanco Á, Moreno-Ger P, Sancho P, Fernández-Manjón B (2012). A narrative metaphor to facilitate educational game authoring. Comput. Educ..

[54] Luctkar-Flude M, Tyerman J, Verkuyl M (2021). Effectiveness of debriefing methods for virtual simulation: A systematic review. Clin. Simulat. Nursing..

[55] Joyner B, Young L (2006). Teaching medical students using role play: Twelve tips for successful role plays. Med. Teach..

[56] Csikszentmihalyi M (1990). Flow: The Psychology of Optimal Performance.

[57] Buajeeb W, Chokpipatkun J, Achalanan N, Kriwattanawong N, Sipiyaruk K (2023). The development of an online serious game for oral diagnosis and treatment planning: Evaluation of knowledge acquisition and retention. BMC Med. Educ..

[58] Littlefield JH, Hahn HB, Meyer AS (1999). Evaluation of a role-play learning exercise in an ambulatory clinic setting. Adv. Health. Sci. Educ. Theory Pract..

[59] Alkin MC, Christie CA (2002). The use of role-play in teaching evaluation. Am. J. Evaluat..

[60] Lovell KL, Mavis BE, Turner JL, Ogle KS, Griffith M (1998). Medical students as standardized patients in a second-year performance-based assessment experience. Med. Educ. Online..

