# Supplementary material for: The design and evaluation of gamified online role-play as a telehealth training strategy in dental education: an explanatory sequential mixed-methods study
Source: Sci Rep. 2024 Apr 22;14:9216. doi: 10.1038/s41598-024-58425-9 (PMC11035619; doi:10.1038/s41598-024-58425-9)
Supplement: Supplementary file 1 — Supplementary Information 1. [file 41598_2024_58425_MOESM1_ESM.pdf]

User satisfactions toward the implementation of gamified online role-play for training teledentistry

Part 1: Self-perceived usefulness toward the gamified online role-play

Please rate each of the following items based on your perceptions toward the usefulness of gamified online role-play for training teledentistry

Perceptions	1 Strongly disagree	2 Disagree	3 Neither agree or disagree	4 Agree	5 Strongly agree
The gamified online role-play enhanced my communication skill with patients using video teleconference.	☐	☐	☐	☐	☐
The gamified online role-play was effective in providing a better picture of the use of teledentistry.	☐	☐	☐	☐	☐
The gamified online role-play promoted my critical thinking and reflective skills.	☐	☐	☐	☐	☐
The gamified online role-play allowed me to identify potential problems while communicating with patients through teledentistry.	☐	☐	☐	☐	☐
The gamified online role-play developed my problem-solving skills associated to teledentistry.	☐	☐	☐	☐	☐
The gamified online role-play was supportive in helping me identify my role in providing dental care using teledentistry.	☐	☐	☐	☐	☐

Part 2: Self-perceived ease of use toward the gamified online role-play

Please rate each of the following items based on your perceptions toward the ease of use of gamified online role-play for training teledentistry

Perceptions	1 Strongly disagree	2 Disagree	3 Neither agree or disagree	4 Agree	5 Strongly agree
I was able to access learning activity of the gamified online role-play with flexibility in location.	☐	☐	☐	☐	☐
I could use my available device (laptop or smartphone) to participate in the gamified online role-play.	☐	☐	☐	☐	☐
The navigation through the gamified online role-play was straightforward.	☐	☐	☐	☐	☐
The storytelling in the gamified online role-play was clear and easy to follow.	☐	☐	☐	☐	☐
Learning sequence presented in the gamified online role-play was appropriate.	☐	☐	☐	☐	☐
Learning time allocated for the gamified online role-play was appropriate.	☐	☐	☐	☐	☐

Part 3: Self-perceived enjoyment toward the implementation of teledentistry

Please rate each of the following items based on your perceptions toward the enjoyment of gamified online role-play for training teledentistry

Perceptions	1<br>Strongly disagree	2<br>Disagree	3<br>Neither agree or disagree	4<br>Agree	5<br>Strongly agree
The game elements of the gamified online role-play motivated me to learn teledentistry.	☐	☐	☐	☐	☐
The format of the gamified online role-play kept me engaged with the learning task for an extended period.	☐	☐	☐	☐	☐
The storytelling of the role-play patient made me enjoy learning teledentistry.	☐	☐	☐	☐	☐
Facial expression and voice tone exhibited from the role-play patient made me enjoy learning teledentistry.	☐	☐	☐	☐	☐
The challenges presented in the gamified online role-play made me feel more excited while learning teledentistry.	☐	☐	☐	☐	☐
I would like to participate in the gamified online role-play for a longer period with other learning scenarios.	☐	☐	☐	☐	☐
