# Supplementary material for: The design and evaluation of gamified online role-play as a telehealth training strategy in dental education: an explanatory sequential mixed-methods study
Source: Sci Rep. 2024 Apr 22;14:9216. doi: 10.1038/s41598-024-58425-9 (PMC11035619; doi:10.1038/s41598-024-58425-9)
Supplement: Supplementary file 2 — Supplementary Information 2. [file 41598_2024_58425_MOESM2_ESM.pdf]

Perceptions toward the implementation of teledentistry in dental practice

---

Part 1: Self-perceived confidence toward the implementation of teledentistry

Please rate each of the following items based on your perceptions toward your confidence in implementing teledentistry.

Perceptions	1 Strongly disagree	2 Disagree	3 Neither agree or disagree	4 Agree	5 Strongly agree
I understand my role in using teledentistry within dental service.	☐	☐	☐	☐	☐
I possess knowledge of the methods to access teledentistry in my dental practice.	☐	☐	☐	☐	☐
I possess knowledge of the procedures for implementing teledentistry in my dental practice.	☐	☐	☐	☐	☐
I am able to use teledentistry for patient care in my dental practice.	☐	☐	☐	☐	☐
I am able to explain the procedures for implementing teledentistry to other staff members.	☐	☐	☐	☐	☐
I have awareness of potential concerns associated with the implementation of teledentistry.	☐	☐	☐	☐	☐

Part 2: Self-perceived awareness toward the implementation of teledentistry

Please rate each of the following items based on your perceptions toward your awareness in implementing teledentistry.

Perceptions	1 Strongly disagree	2 Disagree	3 Neither agree or disagree	4 Agree	5 Strongly agree
Teledentistry enables me to gather comprehensive patient information, thereby facilitating treatment planning in dental services.	☐	☐	☐	☐	☐
Teledentistry can provide additional access options to dental services, especially for individuals encountering travel restrictions, such as patients in remote areas or with limiting mobility.	☐	☐	☐	☐	☐
Teledentistry results in time and cost savings to dental services, compared to in-office appointments.	☐	☐	☐	☐	☐
Teledentistry has the potential for patient follow-up and monitoring in dental services.	☐	☐	☐	☐	☐
Teledentistry can serve as a tool for conducting initial symptom assessments and emergency management in dental care.	☐	☐	☐	☐	☐
Teledentistry can mitigate strain and crowding in dental services, reducing congestion.	☐	☐	☐	☐	☐
